# Impact of anti-thymocyte globulin on survival outcomes in female-to-male allogeneic hematopoietic stem cell transplantation

**DOI:** 10.1038/s41598-023-34442-y

**Published:** 2023-05-03

**Authors:** Masaharu Tamaki, Yu Akahoshi, Masahiro Ashizawa, Yukiko Misaki, Satoshi Koi, Sung-Won Kim, Yukiyasu Ozawa, Shin-ichiro Fujiwara, Shinichi Kako, Ken-ichi Matsuoka, Masashi Sawa, Yuta Katayama, Makoto Onizuka, Yoshinobu Kanda, Takahiro Fukuda, Yoshiko Atsuta, Kimikazu Yakushijin, Hideki Nakasone

**Affiliations:** 1 Division of Hematology, Jichi Medical University Saitama Medical Center, 1-847 Amanuma-cho, Omiya-ku, Saitama, 330-8503 Japan; 2 Tisch Cancer Institute, Icahn School of Medicine at Mount Sinai, New York, USA; 3 Division of Hematology, Jichi Medical University, Shimotsuke, Japan; 4 Hematology Division, Tokyo Metropolitan Cancer and Infectious Disease Center, Komagome Hospital, Tokyo, Japan; 5 Hematopoietic Stem Cell Transplantation Division, National Cancer Center Hospital, Tokyo, Japan; 6 Department of Hematology, Japanese Red Cross Aichi Medical Center Nagoya Daiichi Hospital, Nagoya, Japan; 7 Department of Hematology and Oncology, Okayama University Hospital, Okayama, Japan; 8 Department of Hematology and Oncology, Anjo Kosei Hospital, Anjo, Japan; 9 Department of Hematology, Hiroshima Red Cross Hospital & Atomic-Bomb Survivors Hospital, Hiroshima, Japan; 10 Department of Hematology/Oncology, Tokai University School of Medicine, Isehara, Japan; 11 Japanese Data Center for Hematopoietic Cell Transplantation, Nagakute, Japan; 12 Department of Registry Science for Transplantation and Cellular Therapy, Aichi Medical University School of Medicine, Nagakute, Japan; 13 Department of Medical Oncology and Hematology, Kobe University Hospital, Kobe, Japan; 14 Division of Stem Cell Regulation, Center for Molecular Medicine, Jichi Medical University, 3311-1 Yakushiji, Shimotsuke, 329-0498 Japan

**Keywords:** Haematological cancer, Outcomes research, Haematological diseases

## Abstract

Allogeneic hematopoietic cell transplantation between female donors and male recipients (female-to-male allo-HCT) is a well-established risk factor for inferior survival outcomes due to a higher incidence of graft-versus-host disease (GVHD). However, a clinical significance of anti-thymocyte globulin (ATG) in the female-to-male allo-HCT has not been elucidated. In this study, we retrospectively evaluated male patients who underwent allo-HCT between 2012 and 2019 in Japan. In the female-to-male allo-HCT cohort (n = 828), the use of ATG was not associated with a decreased risk of GVHD (HR of acute GVHD 0.691 [95% CI: 0.461–1.04], *P* = 0.074; HR of chronic GVHD 1.06 [95% CI: 0.738–1.52], *P* = 0.76), but was associated with favorable overall survival (OS) and a decreased risk of non-relapse mortality (NRM) (HR of OS 0.603 [95% CI: 0.400–0.909], *P* = 0.016; HR of NRM 0.506 [95% CI: 0.300–0.856], *P* = 0.011). The use of ATG in female-to-male allo-HCT resulted in survival outcomes that were almost equivalent to those in the male-to-male allo-HCT group. Therefore, GVHD prophylaxis with ATG might overcome the inferiority of survival outcomes in female-to-male allo-HCT.

## Introduction

Graft-versus-host disease (GVHD) is one of the most common complications after allogeneic hematopoietic cell transplantation (allo-HCT) and causes high morbidity and mortality^1–3^. Several efforts have been made to overcome the adverse impact of GVHD on survival outcomes^2^. In vivo T cell depletion with anti-thymocyte globulin (ATG) is a widely accepted procedure for GVHD prophylaxis. Several previous reports have shown a relationship between ATG and a reduced incidence of GVHD^4–7^. However, the immunosuppressive effect of ATG raises a concern about higher incidences of infectious complications and relapse, and the clinical impact of ATG on relapse, infection and overall survival is still a matter of debate.

Allo-HCT between female donors and male recipients (female-to-male allo-HCT) is a well-established risk factor for inferior survival outcomes^8,9^. Since male recipients have H-Y antigens, which are proteins encoded by the Y-chromosome and thus specific to males, these proteins are potential targets of GVHD in female-to-male allo-HCT, resulting in a higher incidence of chronic GVHD and non-relapse mortality (NRM)^10,11^. It may be reasonable to try to improve the survival inferiority of female-to-male allo-HCT by intensive GVHD prophylaxis. However, since previous reports have suggested a relationship between H-Y antigens and a graft-versus leukemia (GVL) effect^10,12^, an escalation of GVHD prophylaxis might induce an increased incidence of relapse in addition to infectious complications. We hypothesized that the use of ATG in female-to-male allo-HCT may contribute to a lower incidence of chronic GVHD and a higher incidence of relapse, and the prognosis in female-to-male allo-HCT may depend on which of these effects is predominant. Therefore, it is important to clarify an adequate GVHD prophylaxis for female-to-male allo-HCT. In this study, we evaluated the impact of ATG on survival outcomes in female-to-male allo-HCT.

## Patients and methods

### Data source and patient selection

Clinical data were obtained from the Transplant Registry Unified Management Program (TRUMP) of the Japanese Society for Transplantation and Cellular Therapy (JSTCT) and the Japanese Data Center for Hematopoietic Cell Transplantation (JDCHCT)^13,14^. This retrospective study was approved by the data management committee of JSTCT (approved number: WG20-64) and by the Institutional Review Board of Jichi Medical University Saitama Medical Center (approved number: S21-122), and performed in accordance with the Declaration of Helsinki and its later amendments. Written informed consent was obtained from all participants in this study.

This study included both adult and pediatric male recipients who were diagnosed as a standard risk disease status and who received their first allo-HCT from an unrelated human leukocyte antigen (HLA)-matched (8/8) or 1-locus mismatched (7/8) donor between January 2012 and December 2019 in Japan. Donor sources were limited to bone marrow (BM) or peripheral blood (PB). This study included acute myeloid leukemia (AML) in complete remission (CR 1, CR 2, CR 3-), acute lymphoblastic leukemia (ALL) in complete remission (CR 1, CR 2, CR 3-), myelodysplastic syndrome (MDS), chronic myeloid leukemia (CML) in chronic phase (CP 1, CP 2, CP 3-) and accelerated phase (AP), myeloproliferative neoplasm (MPN), malignant lymphoma (ML) in complete remission (CR 1, CR 2, CR 3-) including adult T cell leukemia/lymphoma (ATL). We excluded patients who were diagnosed with other diseases and for whom information on gender, survival and the last follow-up date were not available.

### Definitions

Disease risk index (DRI) was classified according to a previous report^15^. With regard to cytogenetics of DRI, for AML, t(8;21), inv(16) and t(15;17) were classified as favorable, complex (≥ 4 abnormalities) was classified as adverse, and other karyotypes were classified as intermediate. For MDS, abnormal chromosome 7 and complex karyotype (≥ 4 abnormalities) were classified as adverse and the other karyotypes were considered intermediate. ATL was classified as high risk in DRI. The hematopoietic cell transplantation comorbidity index (HCT-CI) was assessed based on a previous report^16^. Conditioning regimens were classified according to the criteria from the Center for International Blood and Marrow Transplantation Research^17^. Briefly, myeloablative conditioning regimen (MAC) was defined as total body irradiation (TBI) > 8 Gy (fractionated) or > 5 Gy (single dose), melphalan > 140 mg/m^2^, intravenous busulfan > 7.2 mg/kg, and all other regimens were classified as reduced-intensity conditioning (RIC). Chronic GVHD was diagnosed and graded based on standard criteria^18^.

### Statistical analysis

First, survival outcomes were compared between the female-to-male and male-to-male allo-HCT groups to validate the adverse impact of female-to-male allo-HCT in the entire cohort. Second, survival outcomes were compared between patients who received GVHD prophylaxis with and without ATG in the female-to-male allo-HCT cohort. Third, the impact of ATG was similarly analyzed in the male-to-male allo-HCT cohort. Subsequently, the effect of ATG was discussed based on the results in both the female-to-male and male-to-male allo-HCT cohort.

Categorical and continuous variables were compared by Fisher’s exact test and the Mann–Whitney U test, respectively. Overall survival (OS) was estimated by the Kaplan–Meier method and compared by the log-rank test. Cumulative incidences of relapse, NRM, and acute and chronic GVHD were estimated by Gray’s test. Relapse and NRM were treated as competing risks for each other. Death due to any cause was treated as a competing risk for acute and chronic GVHD. To adjust for the effect of early death, which can inappropriately lower the incidence of chronic GVHD, the cumulative incidence of chronic GVHD was evaluated among patients who survived without disease relapse for more than 100 days after allo-HCT. A Cox proportional hazard regression model was used for multivariate analyses of survival outcomes. Our interest of research questions was to confirm the adverse impact of sex-mismatched HCT and to pursue the effect of ATG in the limited cohort of sex-mismatched HCT. There were various confounding factors which affects donor selection and use of ATG. To address the potential confounding factors and ensure accurate results, multivariate analysis was performed in all survival analyses, regardless of the results of univariate analyses. Thus, the hazard ratios (HR) of types of sex-mismatched HCT or ATG were adjusted for the following pre-transplant clinical factors: age, disease type, DRI, HCT-CI, donor type and source, conditioning regimen, GVHD prophylaxis and allo-HCT year. A two-tailed *P* value < 0.05 was considered to be statistically significant. All analyses in this study were performed with EZR (http://www.jichi.ac.jp/saitama-sct/SaitamaHP.files/statmedEN.html, Jichi Medical University Saitama Medical Center, Saitama, Japan), which is a graphical user interface for R version 3.6.3 (The Foundation for Statistical Computing, Vienna, Austria)^19^.

## Results

### Patient characteristics

According to the eligibility criteria, 828 female-to-male allo-HCT patients and 2431 male-to-male allo-HCT patients were identified. The median follow-up duration for survivors was 39.2 months (range, 0.1–105.5) in the entire cohort, and was shorter in the ATG group (26.2 months in the ATG group vs 41.2 months in the non-ATG group, *P* < 0.001). The median age was 51 years (range, 0–75) in the female-to-male cohort, and 52 years (range, 0–74) in the male-to-male cohort. Out of the patients for whom we had information regarding ATG (n = 413), thymoglobulin (ATG-T, Sanofi, Paris, France) was administered to 408 patients, while anti-human T lymphocyte immunoglobulin (ATG-F, Fresenius Biotech GmbH, Munich, Germany) was administered to 4 patients. The information on ATG administration was unavailable for one patient. The median dose of ATG-T was 2.5 mg/kg (inter quartile range: 1.5–2.5). Among patients whose information about chronic GVHD severity according to NIH criteria^20^ was available (n = 2325), the ATG group tended to include fewer patients who were suffered from severe chronic GVHD compared with the non-ATG group (2 cases [0.6%] vs 45 cases [2.3%], *P* = 0.055). Patient characteristics are shown in Supplemental Table S1. The female-to-male cohort included more patients who were younger than 16 years old (79 cases [9.5%] vs 105 cases [4.3%], *P* < 0.001). The other characteristics were almost equivalent between these cohorts.

### Confirmation of the adverse impact of female-to-male allo-HCT on survival outcomes

When survival outcomes were simply compared between the female-to-male and male-to-male allo-HCT groups, OS tended to be inferior to that for male-to-male allo-HCT (5-y OS 55.2% vs 59.4%, *P* = 0.11), and NRM and chronic GVHD of female-to-male allo-HCT tended to be higher than those of male-to-male allo-HCT (5-y NRM 28.2% vs 24.2%, *P* = 0.066; 5-y cGVHD 48.0 vs 42.8%, *P* = 0.059). Multivariate analyses revealed that female-to-male allo-HCT was significantly associated with the risk of inferior OS (HR 1.16 [95% confidence interval: 1.02–1.33], *P* = 0.027), and increased NRM (HR 1.20 [95% CI: 1.02–1.42], *P* = 0.030) and chronic GVHD (HR 1.19 [95% CI: 1.04–1.36], *P* = 0.010). On the other hand, the cumulative incidence of relapse (CIR) in female-to-male allo-HCT was not different from that in male-to-male allo-HCT (5-y CIR 19.3% vs 19.7%, *P* = 0.71; HR 0.996 [95% CI: 0.821–1.21], *P* = 0.97) (Supplemental Table S2). Regarding acute GVHD, grade II–IV acute GVHD (33.9% vs 40.8%, *P* < 0.001) seemed to be less common in female-to-male allo-HCT due to the greater mortality before the onset of acute GVHD in the current female-to-male allo-HCT cohort (27.8% vs 21.5%, *P* < 0.001).

### Clinical impact of ATG in the female-to-male allo-HCT cohort

In the female-to-male allo-HCT cohort (n = 828), 117 patients received ATG as GVHD prophylaxis. The ATG group included more patients who underwent allo-HCT from an HLA mismatched donor, more patients who used PB as a donor source, more patients who received RIC as a conditioning regimen, and more patients who underwent allo-HCT after 2015 (Table 1).

**Table 1:** Patient characteristics.

	Female-to-male allo-HCT cohort			Male-to-male allo-HCT cohort		
	ATG (n = 117)	Non-ATG (n = 711)	*P* value	ATG (n = 314)	Non-ATG (n = 2117)	*P* value
Age						
> 50 years	60 (51.3)	369 (51.9)	0.79	172 (54.8)	1119 (52.9)	0.048
16–50 years	44 (37.6)	276 (38.8)		121 (38.5)	914 (43.2)	
< 16 years	13 (11.1)	66 (9.3)		21 (6.7)	84 (4.0)	
Disease						
AML	34 (29.1)	242 (34.0)	0.21	99 (31.5)	738 (34.9)	0.065
ALL	24 (20.5)	168 (23.6)		58 (18.5)	490 (23.2)	
MDS	43 (36.8)	201 (28.3)		97 (30.9)	586 (27.7)	
CML and MPN	6 (5.1)	58 (8.2)		33 (10.5)	161 (7.6)	
ML including ATL	10 (8.5)	42 (5.9)		27 (8.6)	142 (6.7)	
Disease risk index						
Low	4 (3.4)	42 (5.9)	0.45	17 (5.4)	155 (7.3)	0.083
Intermediate	94 (80.3)	545 (76.7)		248 (79.0)	1579 (74.7)	
High	13 (11.1)	107 (15.0)		36 (11.5)	345 (16.3)	
Very high	0 (0.0)	1 (0.1)		0 (0.0)	1 (0.1)	
HCT-CI						
0–1	96 (82.1)	544 (76.5)	0.23	229 (72.9)	1590 (75.1)	0.33
≥ 2	21 (17.9)	164 (23.1)		85 (27.1)	517 (24.4)	
Donor type						
Matched unrelated	29 (24.8)	440 (61.9)	< 0.001	89 (29.3)	1366 (63.2)	< 0.001
Mismatched unrelated	88 (75.2)	271 (38.1)		225 (71.7)	751 (35.5)	
Donor source						
Bone marrow	91 (77.8)	659 (92.7)	< 0.001	231 (73.6)	1956 (92.5)	< 0.001
Peripheral blood	26 (22.2)	52 (7.3)		83 (26.4)	161 (7.6)	
Conditioning regimen						
MAC	71 (60.7)	505 (71.0)	0.030	196 (62.4)	1496 (70.8)	0.0038
RIC	46 (39.3)	206 (29.0)		118 (37.6)	621 (29.4)	
GVHD prophylaxis						
CsA-based	8 (6.8)	68 (9.6)	0.17	21 (6.7)	167 (7.9)	0.70
TAC-based	104 (88.9)	630 (88.6)		285 (90.8)	1905 (90.1)	
Others	5 (4.3)	13 (1.8)		8 (2.5)	45 (2.1)	
SCT year						
2012–2015	35 (29.9)	381 (53.6)	< 0.001	90 (28.7)	1107 (52.3)	< 0.001
2016–2019	82 (70.1)	330 (46.4)		224 (71.3)	1010 (47.7)	

ATG, anti-thymocyte globulin; AML, acute myeloid leukemia; ALL, acute lymphoblastic leukemia; MDS, myelodysplastic syndrome; CML, chronic myeloid leukemia; MPN, myeloproliferative neoplasm; ML, malignant lymphoma; ATL, adult T cell leukemia/lymphoma; HCT-CI, hematopoietic cell transplantation comorbidity index; MAC, myeloablative conditioning regimen; RIC, reduced intensity conditioning regimen; GVHD, graft-versus-host disease; CsA, cyclosporin; TAC, tacrolimus; SCT, stem cell transplantation.

**Figure 1:**
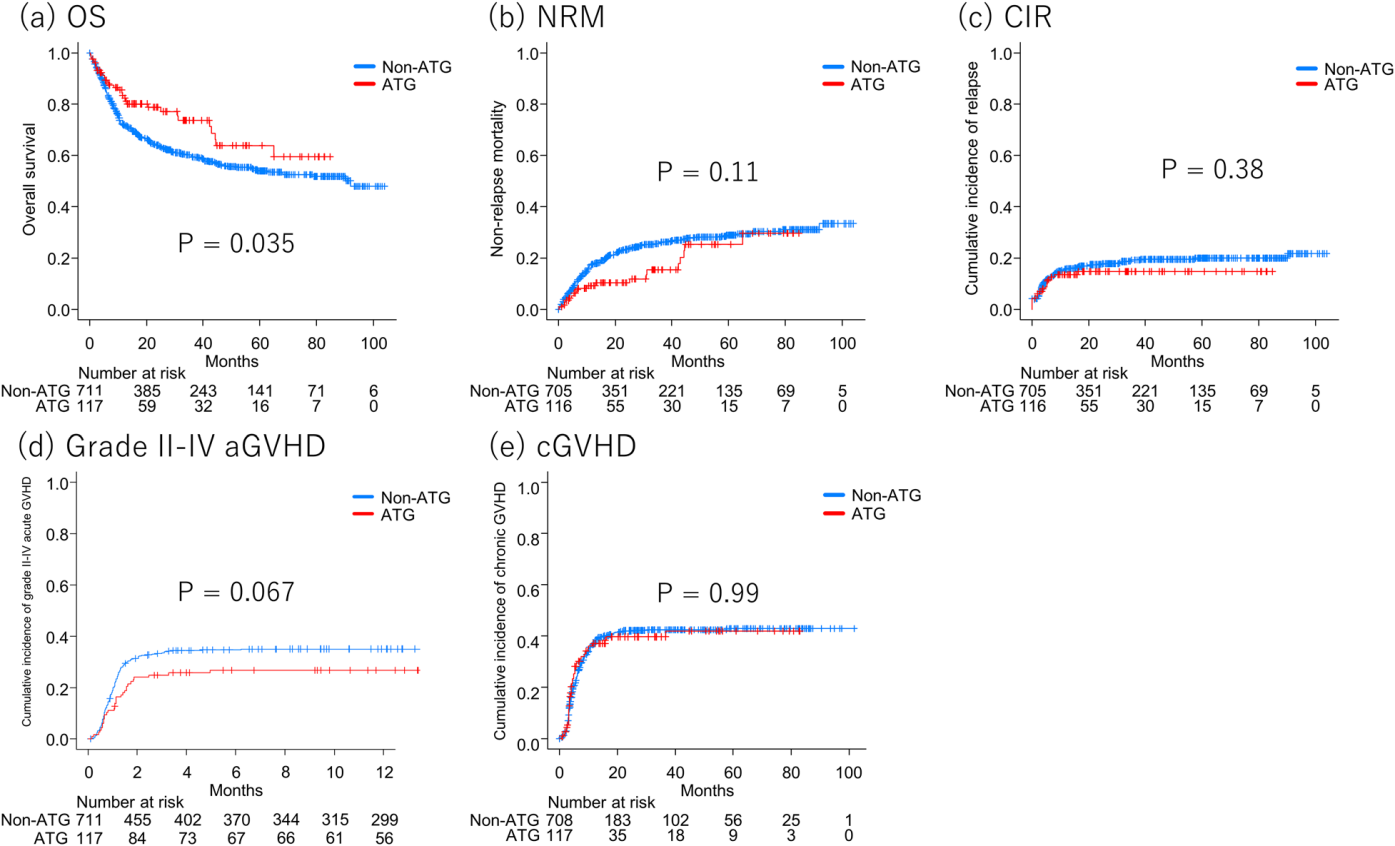
Survival outcomes according to administration of anti-thymocyte globulin in the female-to-male cohort (**a**) Overall survival (**b**) Cumulative incidence of relapse (**c**) Non-relapse mortality (**d**) Cumulative incidence of acute graft-versus-host disease (**e**) Cumulative incidence of chronic graft-versus-host disease.

**Figure 2:**
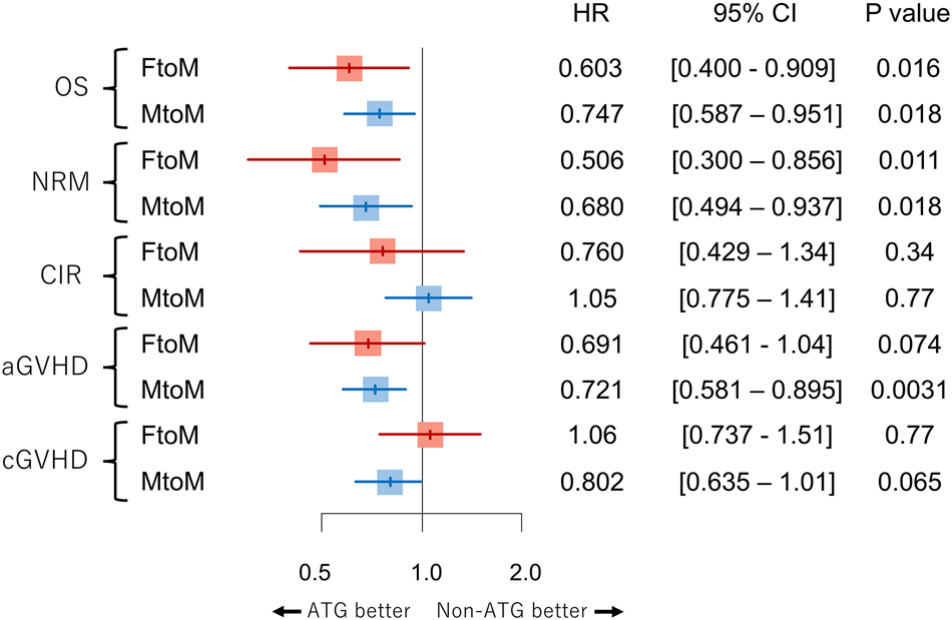
Impact of anti-thymocyte globulin on survival outcomes in the female-to-male (FtoM) and male-to-male (MtoM) allo-HCT cohorts.

The ATG group showed favorable OS compared with the non-ATG group (5-y OS 63.8% vs 53.9%, *P* = 0.035, Fig. 1a). A multivariate analysis also demonstrated that the use of ATG was significantly associated with superior OS (HR 0.603 [95% CI: 0.400–0.909], *P* = 0.016) (Fig. 2, Supplemental Table S3). Similarly, NRM tended to be lower in the ATG group (5-y NRM 25.3% vs 28.9%, *P* = 0.11, Fig. 1b), although the difference was not significant. However, multivariate analysis revealed that the use of ATG was significantly associated with a reduced risk of NRM (HR 0.506 [95% CI: 0.300–0.856], *P* = 0.011). There was no significant difference in CIR between the ATG and non-ATG groups (14.8% vs 19.9%, *P* = 0.38, Fig. 1c), and a multivariate analysis also showed no significant relationship between the use of ATG and CIR (HR 0.760 [95% CI: 0.429–1.34], *P* = 0.34). Grade II–IV acute GVHD tended to be less common in the ATG group (26.7% vs 35.0%, *P* = 0.067, Fig. 1d), and the use of ATG seemed to be associated with a lower incidence of grade II–IV acute GVHD, but this difference was not statistically significant (HR 0.691 [95% CI: 0.461–1.04], *P* = 0.074). The cumulative incidence of chronic GVHD was equivalent between the ATG and non-ATG groups (5-y cumulative incidence of chronic GVHD 47.2% vs 48.2%, *P* = 0.99, Fig. 1e), and ATG was not associated with chronic GVHD in a multivariate analysis (HR 1.06 [95% CI: 0.738–1.52], *P* = 0.76).

### Cause of death in the female-to-male allo-HCT cohort

**Figure 3:**
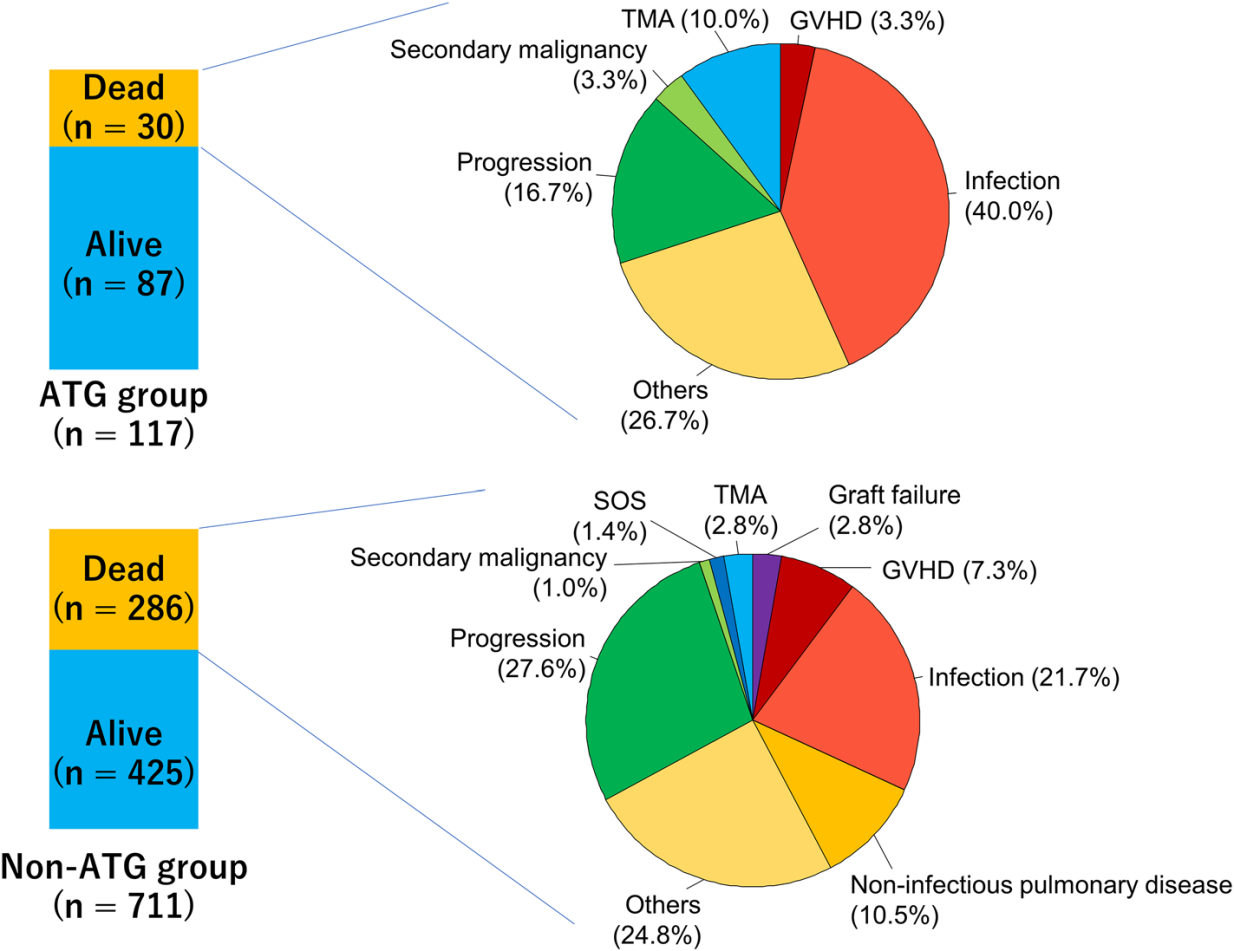
Cause of death in the female-to-male allo-HCT cohort.

The cause of death was mainly infection and disease progression (Fig. 3). The ATG group showed a significantly lower incidence of death due to progression and non-infectious pulmonary complications (progression 5 of 117 cases [4.3%] vs 79 of 711 cases [11.1%], *P* = 0.020; non-infectious pulmonary complications 0 of 117 cases [0.0%] vs 30 of 711 cases [4.2%], *P* = 0.015) (Supplemental Table S4). Although the number of deaths due GVHD was lower in the ATG group, there was no significant difference between the groups (1 of 117 cases [0.9%] vs 21 of 711 cases [3.0%], *P* = 0.35). The other causes of death were comparable between the groups.

### Clinical impact of ATG in the male-to-male allo-HCT cohort

ATG was administered to 314 patients in the male-to-male cohort (n = 2431). The ATG group included more patients who were younger than 16 years old, more patients who underwent allo-HCT from an HLA mismatched donor, more patients who used PB as a donor source, more patients who received RIC as a conditioning regimen and more patients who underwent allo-HCT after 2015 (Table 1).

**Figure 4:**
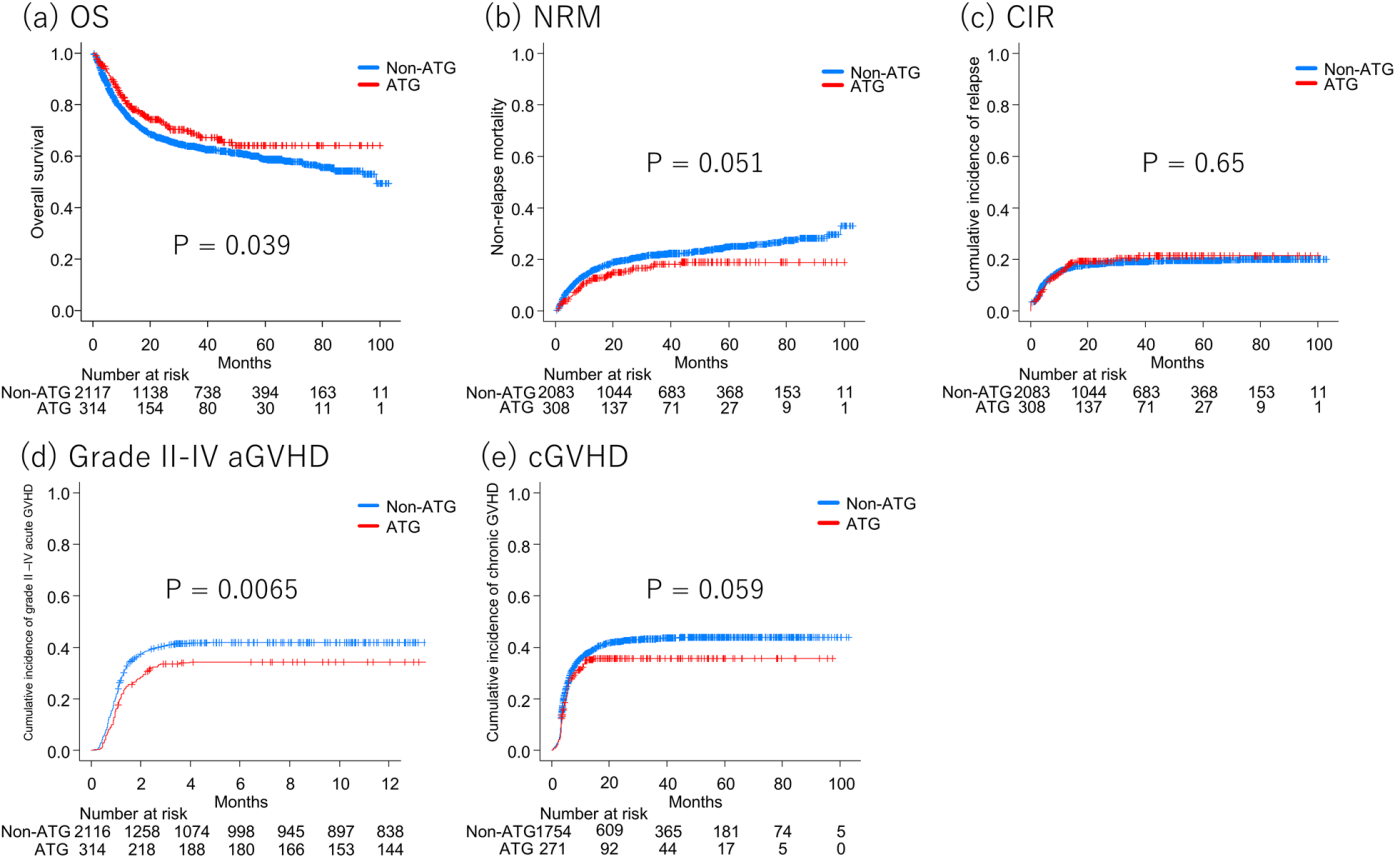
Survival outcomes according to administration of anti-thymocyte globulin in the male-to-male cohort (**a**) Overall survival (**b**) Cumulative incidence of relapse (**c**) Non-relapse mortality (**d**) Cumulative incidence of acute graft-versus-host disease (**e**) Cumulative incidence of chronic graft-versus-host disease.

OS was favorable in the ATG group (5-y OS 64.1% vs 58.8%, *P* = 0.039, Fig. 4a; HR of ATG 0.747 [95% CI: 0.587–0.951], *P* = 0.018, Fig. 2, Supplemental Table S5). The ATG group tended to show lower NRM (5-y NRM 18.9% vs 24.9%, *P* = 0.051, Fig. 4b), and ATG was significantly associated with a reduced risk of NRM (HR 0.680 [95% CI: 0.494–0.937], *P* = 0.018). CIR was comparable between the ATG and non-ATG groups (5-y CIR 21.3% vs 19.4%, *P* = 0.65, Fig. 4c; HR 1.05 [95% CI: 0.775–1.41], *P* = 0.77). The cumulative incidence of grade II–IV acute GVHD was significantly lower in the ATG group (34.2% vs 41.8%, *P* = 0.0065, Fig. 4d), and the use of ATG was associated with a reduced risk of grade II–IV acute GVHD (HR 0.721 [95% CI: 0.581–0.895], *P* = 0.0031).The ATG group tended to show a lower incidence of chronic GVHD (5-y cumulative incidence of chronic GVHD 35.7% vs 43.8%, *P* = 0.059, Fig. 4e), and this tendency was also observed in a multivariate analysis (HR 0.802 [95% CI: 0.635–1.01], *P* = 0.065).

In summary, the impact of ATG seemed to differ somewhat between the female-to-male and male-to-male allo-HCT groups (Fig. 2). The use ATG tended to be associated with a decreased risk of chronic GVHD only in the male-to-male allo-HCT cohort, whereas the impact of ATG on other survival outcomes was almost equivalent between the female-to-male and male-to-male allo-HCT cohorts.

### Survival outcomes stratified according to sex-mismatch and ATG

Additionally, survival analyses were performed in the entire cohort according to the combination of sex-mismatch and ATG. When the female-to-male/ATG group was treated as a reference, the female-to-male/non-ATG group showed an increased risk of poor OS (HR 1.49 [95% CI: 1.03–2.18], *P* = 0.036), whereas the risk of overall mortality was similar to those in both the male-to-male/ATG and male-to-male/non-ATG groups (Supplemental Table S6). An increased risk of NRM also tended to be observed in the female-to-male/non-ATG group (HR 1.51 [95% CI: 0.940–2.42], *P* = 0.089), and the other groups showed a comparable risk of NRM. The risk of chronic GVHD was equivalent among these groups.

## Discussion

In this study, the clinical significance of ATG was evaluated in female-to-male and male-to-male allo-HCT cohorts. In the female-to-male cohort, ATG was significantly associated with favorable OS. Although ATG was not associated with reduced incidences of acute and chronic GVHD in the female-to-male cohort, there was a significant relationship between ATG and favorable NRM. In the male-to-male allo-HCT cohort, ATG was associated with OS and NRM, and also tended to be associated with chronic GVHD.

The sex-mismatched combination of a male recipient and a female donor is recognized as a risk factor for an inferior prognosis in allo-HCT. An unfavorable effect of female-to-male allo-HCT has been reported by several studies since 1989^8,9,21,22^. The sex-mismatched combination is also included in the EBMT score as an unfavorable risk factor^23^. Female-to-male allo-HCT is generally associated with increased incidences of NRM and chronic GVHD. On the other hand, some previous studies showed a reduced incidence of relapse which suggested the existence of a GVL effect due to sex-mismatch^8,24–26^. In fact, we previously reported that male recipients with deletion of Y-chromosome who underwent allo-HCT from a female donor showed a higher incidence of relapse^12^, suggesting attenuation of the GVL effect due to the loss of Y-chromosome. Thus, we initially hypothesized that the addition of ATG may contribute to a lower incidence of chronic GVHD and a higher incidence of relapse in female-to-male allo-HCT.

Based on the hypothesis that female-to-male allo-HCT gives inferior survival outcomes due to a higher incidence of chronic GVHD, it might be reasonable to intensify GVHD prophylaxis in female-to-male allo-HCT. In vivo T cell depletion with ATG is a widely accepted strategy for GVHD prophylaxis. Several randomized control trials have shown that GVHD prophylaxis with ATG was associated with a decreased risk of GVHD and NRM,^4–6^ and various studies have reported the clinical significance of GVHD prophylaxis with ATG^7,27–30^. However, the impact of ATG on OS remains controversial, since the intensification of GVHD prophylaxis with ATG was associated with an increased risk of infectious complications such as cytomegalovirus and Epstein-Barr virus reactivation^31^. Furthermore, intensive immunosuppression with ATG raises a concern about an increase of relapse, although a significant relationship between ATG and relapse has not been elucidated. The current study revealed an improvement of OS and NRM with ATG in female-to-male allo-HCT, even though ATG did not improve the incidence of chronic GVHD.

One potential explanation for the discrepancy between chronic GVHD and OS/NRM might be differences in severity and response to GVHD treatment. Severe or refractory GVHD is generally associated with an increased risk of mortality. Although several studies failed to show the direct adverse effect of chronic GVHD on NRM^32–34^, chronic GVHD was associated with inferior activities of daily living (ADL) and quality of life^35–37^, and severe chronic GVHD is more likely to impair ADL^35^. Since lower ADL is a risk factor for NRM after allo-HCT^38,39^ and all-cause mortality in various situation^40,41^, reducing the risk of severe chronic GVHD could potentially improve NRM. The current study found that the ATG group in the entire cohort tended to include fewer patients with severe chronic GVHD, although the severity in the female-to-male and male-to-male allo-HCT cohort could not be analyzed separately due to small sample size. The lower rate of severe chronic GVHD in the ATG group suggests that the ATG may reduce NRM by alleviating the severity of chronic GVHD even if it does not decrease the incidence of chronic GVHD. Additionally, the ATG group in the female-to-male allo-HCT cohort included fewer patients who died due to non-infectious pulmonary complication which suggests that ATG might reduce the incidence of fatal allogeneic immune reaction. Moreover, CIR was equivalent between the ATG and non-ATG groups in the female-to-male allo-HCT cohort, and ATG was not adversely associated with relapse. Anyway, the true reason for the discrepancy of the ATG impact between GVHD and NRM remains to be elucidated, and further investigation is required for any conclusion.

According to the result of the additional analysis based on a combination of sex-mismatch and ATG, the administration of ATG might reduce the adverse impact of female-to-male allo-HCT and improve OS and NRM, resulting in outcomes comparable to those with male-to-male allo-HCT. For the further improvement of survival outcomes, additional GVHD prophylaxis might be required in female-to-male allo-HCT. A promising approach to GVHD prophylaxis is posttransplant cyclophosphamide (PTCY). PTCY was reported as a highly effective GVHD prophylaxis in previous reports^42–44^. Several studies have shown that PTCY provides superior progression-free survival and GVHD/relapse-free survival compared with ATG^45,46^. Additionally, there have been several attempts to develop a combination therapy with ATG and PTCY^47,48^. Although we should be careful regarding a risk of infection and relapse, further investigation is also warranted to elucidate the clinical significance of PTCY in female-to-male allo-HCT.

The current study has several limitations due to its retrospective nature. First, the number of patients who underwent allo-HCT with ATG has recently increased. Therefore, the median follow-up duration in the ATG group was slightly shorter than that in the non-ATG group. A longer follow-up might reveal additional favorable and/or adverse impacts of ATG. Second, due to the limited sample size, we could not perform various subgroup analyses according to HLA-match/mismatch, donor source or disease type. Third, although the background condition was compensated by DRI and HCT-CI, the use of ATG might be avoided in patients who were at high risk of disease relapse or in poor general condition.

In conclusion, GVHD prophylaxis with ATG in female-to-male allo-HCT was significantly associated with superior OS and a reduced risk of NRM, despite no significant relationship with chronic GVHD. The use of ATG in female-to-male allo-HCT resulted in survival outcomes that were almost the same as those in the male-to-male allo-HCT group. GVHD prophylaxis with ATG might overcome the inferior survival outcomes in female-to-male allo-HCT.

## Supplementary Information

Supplementary Information.

## Data Availability

The datasets analyzed during the current study are available from the corresponding author on reasonable request.
