# Supplementary material for: Impact of anti-thymocyte globulin on survival outcomes in female-to-male allogeneic hematopoietic stem cell transplantation
Source: Sci Rep. 2023 May 3;13:7166. doi: 10.1038/s41598-023-34442-y (PMC10156723; doi:10.1038/s41598-023-34442-y)
Supplement: Supplementary file 1 — Supplementary Information. [file 41598_2023_34442_MOESM1_ESM.pdf]

Impact of anti-thymocyte globulin on survival outcomes in female-to-male allogeneic hematopoietic stem cell transplantation.

Masaharu Tamaki[1] *, Yu Akahoshi[1, 2], Masahiro Ashizawa[3], Yukiko Misaki[1], Satoshi Koi[4], Sung-Won Kim[5], Yukiyasu Ozawa[6], Shin-ichiro Fujiwara[3], Shinichi Kako[1], Ken-ichi Matsuoka[7], Masashi Sawa[8], Yuta Katayama[9], Makoto Onizuka[10], Yoshinobu Kanda[1, 3], Takahiro Fukuda[5], Yoshiko Atsuta[11, 12], Kimikazu Yakushijin[13], Hideki Nakasone[1] *.

1. Division of Hematology, Jichi Medical University Saitama Medical Center, Saitama, Japan.
2. Tisch Cancer Institute, Icahn School of Medicine at Mount Sinai, New York, USA.
3. Division of Hematology, Jichi Medical University, Shimotsuke, Japan.
4. Hematology Division, Tokyo Metropolitan Cancer and Infectious Disease Center, Komagome Hospital, Tokyo, Japan.
5. Hematopoietic Stem Cell Transplantation Division, National Cancer Center Hospital, Tokyo Japan.
6. Department of Hematology, Japanese Red Cross Aichi Medical Center Nagoya Daiichi Hospital, Nagoya, Japan.
7. Department of Hematology and Oncology, Okayama University Hospital, Okayama, Japan.
8. Department of Hematology and Oncology, Anjo Kosei Hospital, Anjo, Japan.
9. Department of Hematology, Hiroshima Red Cross Hospital & Atomic-bomb Survivors Hospital, Hiroshima, Japan.
10. Department of Hematology/Oncology, Tokai University School of Medicine, Isehara, Japan.

11. Japanese Data Center for Hematopoietic Cell Transplantation, Nagakute, Japan.

12. Department of Registry Science for Transplantation and Cellular Therapy, Aichi Medical University School of Medicine, Nagakute, Japan.

13. Department of Medical Oncology and Hematology, Kobe University Hospital, Kobe, Japan.

*** Co-corresponding authors:**

Masaharu Tamaki, M.D. E-mail: m.tamaki.221@gmail.com

Hideki Nakasone, M.D., Ph.D. E-mail: nakasone-tky@outlook.com

Division of Hematology, Jichi Medical University Saitama Medical University Saitama Medical Center,

1-847 Amanuma-cho, Omiya-ku, Saitama city, Saitama 330-8503

TEL: +81-48-647-2111 FAX: +81-48-644-5166

Supplemental Table S1. Patient characteristics in the entire cohort.

		Whole cohort (n = 3259)	FtoM (n = 828)	MtoM (n = 2431)	P value
Age	> 50 years	1720 (52.8)	429 (51.8)	1291 (53.1)	<0.001
	16 – 50 years	1355 (41.6)	320 (38.6)	1035 (42.6)	
	< 16 years	184 (5.6)	79 (9.5)	105 (4.3)	
Disease	AML	1113 (34.2)	276 (33.3)	837 (34.4)	0.91
	ALL	740 (22.7)	192 (23.2)	548 (22.5)	
	MDS	927 (28.4)	244 (29.5)	683 (28.1)	
	CML and other MPN	258 (7.9)	64 (7.7)	194 (8.0)	
	ML (including ATL)	216 (6.6)	52 (6.3)	164 (6.7)	
Disease risk index	Low	218 (6.7)	46 (5.6)	172 (7.1)	0.26
	Intermediate	2466 (75.7)	639 (77.2)	1827 (75.2)	
	High	501 (15.4)	120 (14.5)	381 (15.7)	
	Very high	2 (0.1)	1 (0.1)	1 (0.1)	
HCT-CI	0 – 1	2459 (75.5)	640 (77.3)	1819 (74.8)	0.17
	≥2	787 (24.1)	185 (22.3)	602 (24.8)	
Donor type	Matched unrelated	1924 (59.0)	469 (56.6)	1455 (59.9)	0.11
	Mismatched unrelated	1335 (41.0)	359 (43.4)	976 (40.1)	
Stem cell source	Bone marrow	2937 (90.1)	750 (90.6)	2187 (90.0)	0.64
	Peripheral blood	322 (9.9)	78 (9.4)	244 (10.0)	
Conditioning regimen	MAC	2268 (69.6)	576 (69.6)	1692 (69.6)	1.0
	RIC	991 (30.4)	252 (30.4)	739 (30.4)	
GVHD prophylaxis	CsA-based	264 (8.1)	76 (9.2)	188 (7.7)	0.42
	TAC-based	2924 (89.7)	734 (88.6)	2190 (90.1)	
	Others	71 (2.2)	18 (2.2)	53 (2.2)	
SCT year	2012-2015	1613 (49.5)	416 (50.2)	1197 (49.2)	0.63
	2016-2019	1646 (50.5)	412 (49.8)	1234 (50.8)	
ATG		431 (13.2)	117 (14.1)	314 (12.9)	0.37

Abbreviations: FtoM, female-to-male; MtoM, male-to-male; AML, acute myeloid leukemia; ALL, acute lymphoblastic leukemia; MDS, myelodysplastic syndrome; CML, chronic myeloid leukemia; MPN, myeloproliferative neoplasm; ML, malignant lymphoma; ATL, adult T cell leukemia/lymphoma; HCT-CI, hematopoietic cell transplantation comorbidity index; MAC, myeloablative conditioning regimen; RIC, reduced intensity conditioning regimen; CsA, cyclosporin; TAC, tacrolimus; SCT, stem cell transplantation; ATG, anti-thymocyte globulin.

Supplemental Table S2. Clinical impact of female-to-male in the entire cohort.

		OS		CIR		NRM		Chronic GVHD		Grade II – IV acute GVHD	
		HR (95% CI)	P value	HR (95% CI)	P value	HR (95% CI)	P value	HR (95% CI)	P value	HR (95% CI)	P value
FtoM		1.16 (1.02 - 1.33)	0.027	0.996 (0.821 - 1.21)	0.97	1.20 (1.02 - 1.42)	0.030	1.19 (1.04 - 1.36)	0.010	0.796 (0.695 - 0.911)	<0.001
	> 50 years	Reference	1.0	Reference	1.0	Reference	1.0	Reference	1.0	Reference	1.0
Age	16 – 50 years	0.588 (0.509 - 0.678)	<0.001	0.825 (0.676 - 1.01)	0.060	0.513 (0.428 - 0.616)	<0.001	0.973 (0.848 - 1.12)	0.69	1.07 (0.942 - 1.23)	0.29
	< 16 years	0.286 (0.195 - 0.419)	<0.001	0.618 (0.403 - 0.947)	0.027	0.212 (0.123 - 0.366)	<0.001	0.529 (0.384 - 0.730)	<0.001	1.39 (1.09 - 1.77)	0.0075
	AML	Reference	1.0	Reference	1.0	Reference	1.0	Reference	1.0	Reference	1.0
	ALL	1.23 (1.03 - 1.47)	0.026	1.31 (1.02 - 1.68)	0.036	1.11 (0.887 - 1.39)	0.36	1.09 (0.921 - 1.28)	0.33	0.683 (0.585 - 0.798)	<0.001
Disease	MDS	1.36 (1.14 - 1.64)	<0.001	1.33 (1.03 - 1.73)	0.029	1.31 (1.04 - 1.65)	0.023	1.21 (1.02 - 1.45)	0.034	0.841 (0.716 - 0.988)	0.035
	CML and other MPN	1.54 (1.18 - 2.01)	0.0017	2.31 (1.61 - 3.31)	<0.001	1.33 (0.936 - 1.88)	0.11	1.07 (0.808 - 1.42)	0.63	0.693 (0.540 - 0.890)	0.0041
	ML (including ATL)	1.01 (0.757 - 1.34)	0.96	1.15 (0.768 - 1.73)	0.49	0.940 (0.657 - 1.35)	0.74	1.13 (0.875 - 1.47)	0.34	0.910 (0.718 - 1.15)	0.43
	Low	Reference	1.0	Reference	1.0	Reference	1.0	Reference	1.0	Reference	1.0
Disease risk index	Intermediate	1.47 (1.10 - 1.96)	0.0086	1.99 (1.26 - 3.16)	0.0034	1.19 (0.847 - 1.68)	0.31	1.08 (0.835 - 1.39)	0.57	0.766 (0.606 - 0.969)	0.026
	High and very high	2.57 (1.87 - 3.53)	<0.001	5.32 (3.24 - 8.72)	<0.001	1.54 (1.04 - 2.29)	0.032	1.22 (0.904 - 1.66)	0.19	0.746 (0.564 - 0.986)	0.040
HCT-CI ≥ 2		1.19 (1.04 - 1.36)	0.0092	1.13 (0.937 - 1.37)	0.20	1.18 (0.998 - 1.40)	0.052	0.955 (0.827 - 1.10)	0.54	1.04 (0.907 - 1.18)	0.61
Donor type	Matched unrelated	Reference	1.0	Reference	1.0	Reference	1.0	Reference	1.0	Reference	1.0
	Mismatched unrelated	1.18 (1.04 - 1.33)	0.0085	0.899 (0.752 - 1.08)	0.24	1.34 (1.15 - 1.56)	<0.001	0.982 (0.865 - 1.11)	0.78	1.25 (1.11 - 1.40)	<0.001
Bone marrow		0.990 (0.793 - 1.24)	0.93	0.759 (0.583 - 0.988)	0.040	1.21 (0.879 - 1.65)	0.25	0.780 (0.635 - 0.958)	0.018	1.05 (0.847 - 1.29)	0.68
MAC		1.04 (0.908 - 1.19)	0.58	0.928 (0.766 - 1.12)	0.44	1.02 (0.863 - 1.22)	0.79	1.19 (1.03 - 1.37)	0.022	1.06 (0.925 - 1.21)	0.40
	CsA-based	Reference	1.0	Reference	1.0	Reference	1.0	Reference	1.0	Reference	1.0
GVHD prophylaxis	TAC-based	0.972 (0.787 - 1.20)	0.79	1.16 (0.834 - 1.62)	0.37	0.864 (0.670 - 1.12)	0.26	0.816 (0.664 - 1.00)	0.053	0.941 (0.768 - 1.15)	0.56
	Others	1.46 (0.963 - 2.20)	0.075	1.33 (0.706 - 2.52)	0.37	1.35 (0.800 - 2.29)	0.26	0.957 (0.596 - 1.54)	0.85	1.19 (0.804 - 1.77)	0.38
SCT year	2012-2015	Reference	1.0	Reference	1.0	Reference	1.0	Reference	1.0	Reference	1.0
	2016-2019	0.933 (0.822 - 1.06)	0.28	0.990 (0.832 - 1.18)	0.91	0.938 (0.797 - 1.10)	0.43	0.790 (0.698 - 0.895)	<0.001	0.920 (0.820 - 1.03)	0.16
ATG		0.707 (0.574 - 0.870)	0.0010	0.965 (0.741 - 1.26)	0.79	0.632 (0.481 - 0.830)	<0.001	0.868 (0.714 - 1.06)	0.16	0.720 (0.596 - 0.872)	<0.001

Abbreviations: OS, overall survival; CIR, cumulative incidence of relapse; NRM, non-relapse mortality; GVHD, graft-versus-host disease; HR, hazard ratio; 95% CI, 95% confidence interval; FtoM, female-to-male; AML, acute myeloid leukemia; ALL, acute lymphoblastic leukemia; MDS, myelodysplastic syndrome; CML, chronic myeloid leukemia; MPN, myeloproliferative neoplasm; ML, malignant lymphoma; ATL, adult T cell leukemia/lymphoma; HCT-CI, hematopoietic cell transplantation comorbidity index; MAC, myeloablative conditioning regimen; CsA cyclosporin; TAC, tacrolimus; SCT, stem cell transplantation; ATG, anti-thymocyte globulin.

Supplemental Table S3. Clinical impact of anti-thymocyte globulin in the female-to-male allo-HCT cohort.

		OS		CIR		NRM		Chronic GVHD		Grade II – IV acute GVHD	
		HR (95% CI)	P value	HR (95% CI)	P value	HR (95% CI)	P value	HR (95% CI)	P value	HR (95% CI)	P value
ATG		0.603 (0.400 - 0.909)	0.016	0.760 (0.429 - 1.34)	0.34	0.506 (0.300 - 0.856)	0.011	1.06 (0.737 - 1.51)	0.77	0.691 (0.461 – 1.04)	0.074
Age	> 50 years	Reference	1.0	Reference	1.0	Reference	1.0	Reference	1.0	Reference	1.0
	16 − 50 years	0.422 (0.316 - 0.564)	<0.001	0.801 (0.532 - 1.21)	0.29	0.345 (0.237 - 0.502)	<0.001	0.824 (0.633 - 1.07)	0.15	0.778 (0.553 - 1.10)	0.15
	< 16 years	0.198 (0.102 - 0.383)	<0.001	0.563 (0.276 - 1.15)	0.11	0.185 (0.0791 - 0.435)	<0.001	0.477 (0.297 - 0.767)	0.0022	0.294 (0.139 - 0.623)	0.0014
Disease	AML	Reference	1.0	Reference	1.0	Reference	1.0	Reference	1.0	Reference	1.0
	ALL	1.28 (0.889 - 1.86)	0.18	1.63 (0.964 - 2.74)	0.068	1.09 (0.693 - 1.70)	0.72	0.808 (0.595 - 1.10)	0.17	1.06 (0.698 - 1.61)	0.79
	MDS	1.54 (1.06 - 2.24)	0.025	1.61 (0.926 - 2.80)	0.091	1.38 (0.874 - 2.18)	0.17	1.05 (0.756 - 1.46)	0.77	1.41 (0.908 - 2.19)	0.13
	CML and other MPN	1.73 (1.03 - 2.91)	0.040	3.41 (1.69 - 6.90)	<0.001	1.35 (0.684 - 2.67)	0.39	1.15 (0.696 - 1.91)	0.58	1.11 (0.546 - 2.26)	0.77
	ML (including ATL)	1.26 (0.725 - 2.19)	0.41	0.842 (0.286 - 2.48)	0.75	1.13 (0.590 - 2.18)	0.71	0.733 (0.429 - 1.25)	0.25	1.10 (0.565 - 2.12)	0.79
Disease risk index	Low	Reference	1.0	Reference	1.0	Reference	1.0	Reference	1.0	Reference	1.0
	Intermediate	1.42 (0.801 - 2.53)	0.23	1.66 (0.696 - 3.95)	0.25	1.22 (0.605 - 2.44)	0.58	1.06 (0.633 - 1.77)	0.83	1.02 (0.531 - 1.97)	0.95
	High and very high	2.33 (1.23 - 4.41)	0.0093	4.91 (1.93 - 12.5)	<0.001	1.30 (0.579 - 2.90)	0.53	1.13 (0.616 - 2.07)	0.69	0.875 (0.397 - 1.92)	0.74
HCT-CI ≥ 2		1.24 (0.961 - 1.61)	0.098	1.03 (0.687 - 1.55)	0.88	1.44 (1.05 - 1.98)	0.023	0.739 (0.549 - 0.996)	0.047	0.638 (0.429 - 0.949)	0.026
Donor type	Matched unrelated	Reference	1.0	Reference	1.0	Reference	1.0	Reference	1.0	Reference	1.0
	Mismatched unrelated	1.22 (0.965 - 1.55)	0.096	0.907 (0.632 - 1.30)	0.60	1.59 (1.19 - 2.14)	0.0020	0.916 (0.719 - 1.17)	0.48	0.820 (0.594 - 1.13)	0.23
Bone marrow		0.869 (0.552 - 1.37)	0.54	0.563 (0.328 - 0.966)	0.037	1.12 (0.574 - 2.20)	0.73	0.946 (0.604 - 1.48)	0.81	1.06 (0.583 - 1.94)	0.84
MAC		1.07 (0.833 - 1.38)	0.59	1.36 (0.908 - 2.05)	0.14	0.903 (0.663 - 1.23)	0.52	1.46 (1.11 - 1.92)	0.0071	1.45 (1.01 - 2.07)	0.043
GVHD prophylaxis	CsA-based	Reference	1.0	Reference	1.0	Reference	1.0	Reference	1.0	Reference	1.0
	TAC-based	1.08 (0.735 - 1.57)	0.71	1.80 (0.871 - 3.73)	0.11	0.846 (0.550 - 1.30)	0.45	0.832 (0.576 - 1.20)	0.33	0.765 (0.491 - 1.19)	0.24
	Others	1.89 (0.814 - 4.37)	0.14	1.53 (0.317 - 7.36)	0.60	1.65 (0.609 - 4.45)	0.33	1.21 (0.502 - 2.93)	0.67	0.497 (0.116 - 2.12)	0.34
SCT year	2012-2015	Reference	1.0	Reference	1.0	Reference	1.0	Reference	1.0	Reference	1.0
	2016-2019	0.979 (0.763 - 1.26)	0.87	0.957 (0.660 - 1.39)	0.82	1.01 (0.743 - 1.39)	0.93	0.716 (0.562 - 0.911)	0.0067	0.762 (0.557 - 1.04)	0.089

Abbreviations: OS, overall survival; CIR, cumulative incidence of relapse; NRM, non-relapse mortality; GVHD, graft-versus-host disease; HR, hazard ratio; 95% CI, 95% confidence interval; ATG, anti-thymocyte globulin; AML, acute myeloid leukemia; ALL, acute lymphoblastic leukemia; MDS, myelodysplastic syndrome; CML, chronic myeloid leukemia; MPN, myeloproliferative neoplasm; ML, malignant lymphoma; ATL, adult T cell

leukemia/lymphoma; HCT-CI, hematopoietic cell transplantation comorbidity index; MAC, myeloablative conditioning regimen; CsA cyclosporin; TAC, tacrolimus; SCT, stem cell transplantation.

Supplemental Table S4. Cause of death in the female-to-male allo-HCT cohort.

	ATG (n = 117)	Non-ATG (n = 711)	P value
Progression	5 (4.3)	79 (11.1)	0.020
Infection	12 (10.3)	62 (8.7)	0.60
GVHD	1 (0.9)	21 (3.0)	0.35
Non-infectious pulmonary complication	0 (0.0)	30 (4.2)	0.015
TMA	3 (2.6)	8 (1.1)	0.19
SOS/VOD	0 (0.0)	4 (0.6)	1.0
Graft failure	0 (0.0)	8 (1.1)	0.61
Secondary malignancy	1 (0.9)	3 (0.4)	0.46

Abbreviations: ATG, anti-thymocyte globulin; GVHD, graft-versus-host disease; TMA, thrombotic microangiopathy; SOS/VOD, sinusoidal obstruction syndrome/veno-occlusive disease.

Supplemental Table S5. Clinical impact of anti-thymocyte globulin in the male-to-male allo-HCT cohort.

		OS		CIR		NRM		Chronic GVHD		Grade II – IV acute GVHD	
		HR (95% CI)	P value	HR (95% CI)	P value	HR (95% CI)	P value	HR (95% CI)	P value	HR (95% CI)	P value
ATG		0.747 (0.587 - 0.951)	0.018	1.05 (0.775 - 1.41)	0.77	0.680 (0.494 - 0.937)	0.018	0.802 (0.635 - 1.01)	0.065	0.721 (0.581 - 0.895)	0.0031
Age	> 50 years	Reference	1.0	Reference	1.0	Reference	1.0	Reference	1.0	Reference	1.0
	16 – 50 years	0.659 (0.558 - 0.777)	<0.001	0.843 (0.669 - 1.06)	0.15	0.587 (0.475 - 0.725)	<0.001	1.03 (0.879 - 1.21)	0.69	1.03 (0.886 - 1.19)	0.73
	< 16 years	0.354 (0.221 - 0.567)	<0.001	0.665 (0.386 - 1.14)	0.14	0.229 (0.112 - 0.469)	<0.001	0.508 (0.323 - 0.797)	0.0032	1.47 (1.09 - 1.98)	0.011
Disease	AML	Reference	1.0	Reference	1.0	Reference	1.0	Reference	1.0	Reference	1.0
	ALL	1.20 (0.980 - 1.48)	0.078	1.25 (0.937 - 1.67)	0.13	1.11 (0.854 - 1.44)	0.44	1.23 (1.01 - 1.49)	0.042	0.668 (0.561 - 0.796)	<0.001
	MDS	1.30 (1.05 - 1.61)	0.015	1.30 (0.969 - 1.74)	0.081	1.27 (0.966 - 1.66)	0.087	1.29 (1.05 - 1.60)	0.017	0.801 (0.667 - 0.962)	0.017
	CML and other MPN	1.48 (1.08 - 2.02)	0.015	2.08 (1.37 - 3.17)	<0.001	1.32 (0.876 - 1.98)	0.19	1.05 (0.751 - 1.48)	0.76	0.645 (0.485 - 0.859)	0.0027
	ML (including ATL)	0.934 (0.667 - 1.31)	0.69	1.22 (0.786 - 1.91)	0.37	0.861 (0.557 - 1.33)	0.50	1.31 (0.973 - 1.77)	0.076	0.881 (0.676 - 1.15)	0.35
Disease risk index	Low	Reference	1.0	Reference	1.0	Reference	1.0	Reference	1.0	Reference	1.0
	Intermediate	1.50 (1.08 - 2.10)	0.017	2.13 (1.23 - 3.67)	0.0067	1.21 (0.815 - 1.80)	0.34	1.07 (0.801 - 1.43)	0.64	0.775 (0.595 - 1.01)	0.059
	High and very high	2.70 (1.87 - 3.91)	<0.001	5.53 (3.09 - 9.91)	<0.001	1.67 (1.06 - 2.64)	0.027	1.26 (0.886 - 1.79)	0.20	0.713 (0.519 - 0.978)	0.036
HCT-CI ≥ 2		1.18 (1.01 - 1.38)	0.037	1.15 (0.930 - 1.43)	0.19	1.11 (0.905 - 1.36)	0.32	1.04 (0.878 - 1.22)	0.67	1.09 (0.938 - 1.26)	0.27
Donor type	Matched unrelated	Reference	1.0	Reference	1.0	Reference	1.0	Reference	1.0	Reference	1.0
	Mismatched unrelated	1.17 (1.01 - 1.35)	0.038	0.893 (0.726 - 1.10)	0.28	1.27 (1.06 - 1.53)	0.011	1.00 (0.865 - 1.17)	0.96	1.25 (1.09 - 1.43)	0.001
Bone marrow		1.03 (0.796 - 1.32)	0.84	0.829 (0.611 - 1.12)	0.23	1.22 (0.852 - 1.75)	0.28	0.733 (0.581 - 0.925)	0.0088	1.01 (0.799 - 1.27)	0.95
MAC		1.02 (0.871 - 1.20)	0.80	0.833 (0.668 - 1.04)	0.10	1.07 (0.867 - 1.31)	0.54	1.10 (0.925 - 1.31)	0.28	1.09 (0.930 - 1.27)	0.30
GVHD prophylaxis	CsA-based	Reference	1.0	Reference	1.0	Reference	1.0	Reference	1.0	Reference	1.0
	TAC-based	0.952 (0.737 - 1.23)	0.71	1.02 (0.699 - 1.48)	0.93	0.898 (0.652 - 1.24)	0.51	0.790 (0.616 - 1.01)	0.064	0.844 (0.673 - 1.06)	0.14
	Others	1.38 (0.854 - 2.23)	0.19	1.28 (0.636 - 2.56)	0.49	1.29 (0.691 - 2.42)	0.42	0.887 (0.506 - 1.55)	0.67	1.10 (0.708 - 1.72)	0.66
SCT year	2012-2015	Reference	1.0	Reference	1.0	Reference	1.0	Reference	1.0	Reference	1.0
	2016-2019	0.915 (0.790 - 1.06)	0.24	0.985 (0.807 - 1.20)	0.88	0.922 (0.762 - 1.12)	0.40	0.808 (0.699 - 0.935)	0.0042	0.936 (0.822 - 1.07)	0.32

Abbreviations: OS, overall survival; CIR, cumulative incidence of relapse; NRM, non-relapse mortality; GVHD, graft-versus-host disease; HR, hazard ratio; 95% CI, 95% confidence interval; ATG, anti-thymocyte globulin; AML, acute myeloid leukemia; ALL, acute lymphoblastic leukemia; MDS, myelodysplastic syndrome; CML, chronic myeloid leukemia; MPN, myeloproliferative neoplasm; ML, malignant lymphoma; ATL, adult T cell leukemia/lymphoma; HCT-CI, hematopoietic cell transplantation comorbidity index; MAC, myeloablative conditioning regimen; GVHD, graft-versus-

host disease; CsA, cyclosporin; TAC, tacrolimus; SCT, stem cell transplantation.

Supplemental Table S6. Survival outcomes in subgroups stratified according to sex-mismatch and anti-thymocyte globulin.

	OS		CIR		NRM		Chronic GVHD	
	HR (95% CI)	P value	HR (95% CI)	P value	HR (95% CI)	P value	HR (95% CI)	P value
FtoM/ATG	Reference	1.0	Reference	1.0	Reference	1.0	Reference	1.0
FtoM/nonATG	1.49 (1.03 – 2.18)	0.036	1.33 (0.791 – 2.24)	0.28	1.51 (0.940 – 2.42)	0.089	1.07 (0.751 – 1.43)	0.83
MtoM/ATG	1.05 (0.691 – 1.58)	0.83	1.36 (0.785 – 2.37)	0.27	0.946 (0.556 – 1.61)	0.84	0.736 (0.513 – 1.06)	0.095
MtoM/non-ATG	1.32 (0.916 – 1.90)	0.14	1.32 (0.798 – 2.17)	0.28	1.28 (0.806 – 2.02)	0.30	0.919 (0.676 – 1.25)	0.59

Abbreviations: OS, overall survival; CIR, cumulative incidence of relapse; NRM, non-relapse mortality; GVHD, graft-versus-host disease; HR, hazard ratio; 95% CI, 95% confidence interval; FtoM, female-to-male; MtoM, male-to-male; ATG, anti-thymocyte globulin.
